# Supplementary material for: Tunable magnetless optical isolation with twisted Weyl semimetals
Source: Nanophotonics. 2023 Jul 12;12(16):3333–40. doi: 10.1515/nanoph-2023-0241 (PMC11614334; doi:10.1515/nanoph-2023-0241)
Supplement: Supplementary file 1 — Supplementary Material Details [file j_nanoph-2023-0241_suppl_001.docx]

**Supplemental Material:**

**Tunable Magnetless Optical Isolation with Twisted Weyl Semimetals**

Vladislav Chistyakov^1^, Viktar S. Asadchy^2^, Shanhui Fan^3^, Andrea Alù^4^, and Alex Krasnok^5,*^

*^1^Saint-Petersburg, 191002, Russia*

*^2^Department of Electronics and Nanoengineering, Aalto University, 02150, Espoo, Finland*

*^3^Ginzton Laboratory and Department of Electrical Engineering, Stanford University, Stanford, CA, 94305, USA*

*^4^Photonics Initiative, Advanced Science Research Center, City University of New York, New York, NY, USA*

*^5^Department of Electrical and Computer Engineering, Florida International University, Miami, FL 33174, USA*

*^*^e-mail:* akrasnok@fiu.edu

**Optical properties of used Weyl semimetals**

The optical properties of Weyl semimetals (WS) have been studied theoretically in various papers, see, e.g., Refs. [1,2]. In these works, it has been demonstrated that to account for WS topological properties in the optical response, one may use the standard form of Maxwell equations with $\mathbf{D}=\hat{\varepsilon}_{WS}\mathbf{E}$ and the WS relative permittivity tensor [1,2]

$\hat{\varepsilon}_{WS}=\left(\begin{matrix} \varepsilon_{d} & i\varepsilon_{a} & 0 \\ -i\varepsilon_{a} & \varepsilon'_{d} & 0 \\ 0 & 0 & \varepsilon_{d} \end{matrix}\right)$ (S1)

and unity magnetic permeability. The off-diagonal components are caused by the Weyl nodes splitting in the momentum space by the vector $\mathbf{b}$. They are responsible for the strength of magneto-optical activity and breaking the Lorentz reciprocity. The diagonal components of the in-plane isotropic WSs can be calculated using the Kubo-Greenwood formalism in the approximation of random phases to the two-band model with spin degeneracy  [2,3]:

$\varepsilon_{d}=\varepsilon_{0}\varepsilon_{b}+\frac{ir_{s}g\varepsilon_{0}}{6\mathrm{Re}(\Omega)}\Omega G(\frac{E_{F}\Omega}{2})-\frac{r_{s}g\varepsilon_{0}}{6\pi\mathrm{Re}(\Omega)}\left\{\frac{4}{\Omega}\left[1+\frac{\pi^{2}}{3}\left(\frac{k_{B}T}{E_{F}(T)}\right)^{2}\right]+8\Omega\int_{0}^{\xi_{c}} \frac{G(E_{F}\xi)-G(\frac{E_{F}\Omega}{2})}{\Omega^{2}-4\xi^{2}}\xi d\xi\right\}$ (S2)

where $\varepsilon_{b}$ is the background permittivity, $E_{F}$ is the chemical potential, is the normalized frequency, $\tau^{-1}$ is the Drude damping rate, $G(E)=n(-E)-n(E)$, where $n(E)$ is the Fermi distribution function, is the effective fine-structure constant, $\nu_{F}$ is the Fermi velocity, *g* is the number of Weyl points, and $\xi_{c}=E_{c}/E_{F}$, where $E_{c}$ is the cutoff energy beyond which the band dispersion is no longer linear. For the real part of the equation (S2), according to [3], we obtain

$\text{Re }\varepsilon_{d}(\Omega)=\varepsilon_{\infty}(1-\Omega_{p}^{2}/\Omega^{2})$, (S3)

where $\Omega_{p}^{2}=2r_{s}g/(3\pi\varepsilon_{\infty})$ is the plasma frequency and $\varepsilon_{\infty}$ is the effective background dielectric constant. In our work, the anisotropic component of the dielectric tensor is determined by $\varepsilon_{d}^{'}(\Omega_{p})=\varepsilon_{d}(q\Omega_{p})$, where *q* plasma frequency offset coefficient.

We used the following parameters [1] $\varepsilon_{b}=6.2$, $\xi_{c}=3$, $\tau=1000$ fs, $g=2$, $b=8.5\times10^{8}$m^-1^, $\nu_{F}=0.83\times10^{5}$ m s^-1^, $E_{F}=0.3$ eV at $T=300$ K, and $q=0.68$. **Fig. S1** shows the frequency dispersion of the real part of the $\varepsilon_{d}$, $\varepsilon_{a}$, and $\varepsilon_{d}^{'}$ components. The figure shows that almost in the entire frequency range $\varepsilon_{a}$ and $\varepsilon_{d}$ are of the same order, which indicates a high value of the magneto-optical parameter $\varepsilon_{a}/\varepsilon_{d}\approx1$, which is significantly higher than for conventional magneto-optical materials [4]. Also, an additional plasma resonance appears in the material corresponding to the dotted blue line due to anisotropy.


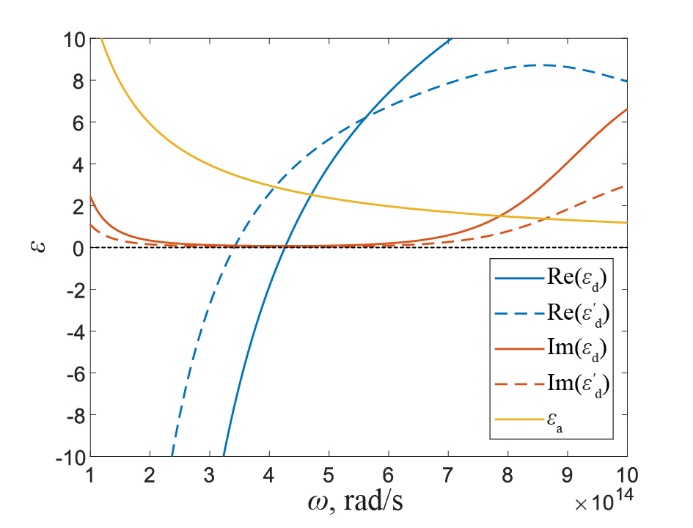


**Fig. S1**. Permetivity tensor components dispersion of the anisotropic Weyl semimetal.

**Polarizer-less optical isolator with WSs**

The anisotropy of WSs in this geometry can operate as a polarizer, eliminating the traditional polarizer and analyzer and making the design even more compact. **Fig. S2(a)** shows the geometry of the proposed polarizer-less compact isolator. In this configuration, we consider the transmission of only cross-polarizations $T_{+z}^{yx}/T_{-z}^{xy}$. The thickness of the WS is adjusted to $h_{W}=0.46$ μm to reach a maximum asymmetry $I_{1}=T_{+z}^{yx}-T_{-z}^{xy}$ of 16%, **Fig. S2(b).** The maximum isolation for selected optimized parameters reaches 50 dB at the frequency $\omega=3.03\times10^{14}$ rad/s, **Fig. S2(c)**. Other geometrical parameters are the same as in **Figs. 1,2** in the main text. The results of the isolation spectrum as a function of the relative rotation is shown in **Fig. S2(d)**. The maximum reaches 60 dB at $\Psi=-20^{\circ}$ and at frequency $\omega=3.05\times10^{14}$ rad/s. The isolator can operate in both directions by adjusting the twist angle.


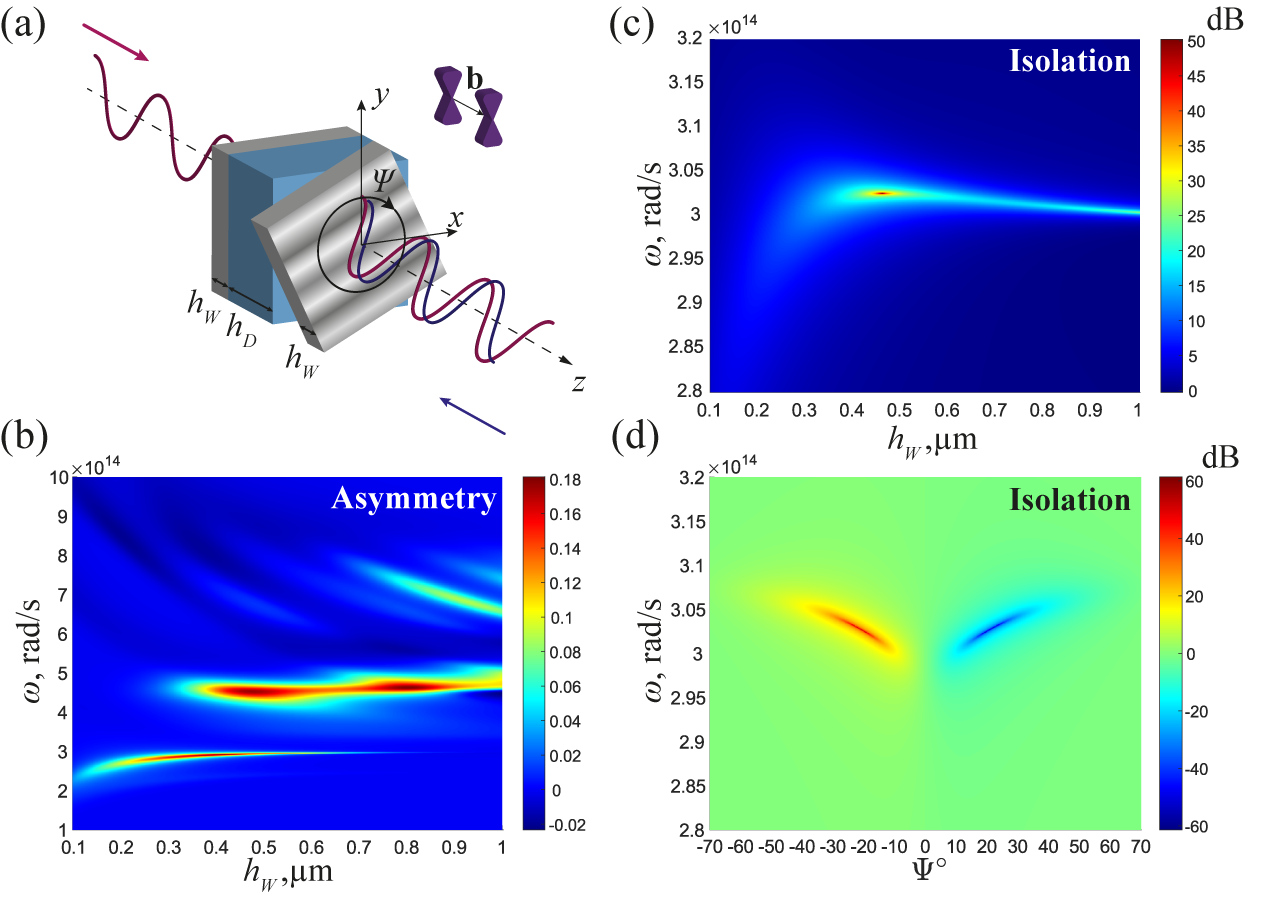


**Fig. S2.** (a) Isolator geometry without polarizers. (b),(c) Asymmetry $\Delta T=|T_{+z}^{yx}|^{2}-|T_{-z}^{xy}|^{2}$ and isolation $I=10\lg\left(|T_{+z}^{yx}|^{2}/|T_{-z}^{xy}|^{2}\right)$ spectrum as a function of frequency and thickness of the Weyl semimetal at the rotation angle $\Psi=-20^{\circ}$. (d) Isolation as a function of frequency and angle of rotation.

**References**

[1] O. V. Kotov and Y. E. Lozovik, *Giant Tunable Nonreciprocity of Light in Weyl Semimetals*, Phys. Rev. B **98**, 195446 (2018).

[2] J. Hofmann and S. Das Sarma, *Surface Plasmon Polaritons in Topological Weyl Semimetals*, Phys. Rev. B **93**, 241402 (2016).

[3] O. V. Kotov and Y. E. Lozovik, *Dielectric Response and Novel Electromagnetic Modes in Three-Dimensional Dirac Semimetal Films*, Phys. Rev. B **93**, 1 (2016).

[4] V. S. Asadchy, M. S. Mirmoosa, A. Diaz-Rubio, S. Fan, and S. A. Tretyakov, *Tutorial on Electromagnetic Nonreciprocity and Its Origins*, Proc. IEEE **108**, 1684 (2020).
